# Herd-level risk factors associated with the presence of Phage type 21/28 *E. coli *O157 on Scottish cattle farms

**DOI:** 10.1186/1471-2180-6-99

**Published:** 2006-12-02

**Authors:** Jo EB Halliday, Margo E Chase-Topping, Michael C Pearce, Iain J McKendrick, Lesley Allison, Dave Fenlon, Chris Low, Dominic J Mellor, George J Gunn, Mark EJ Woolhouse

**Affiliations:** 1 Centre for Infectious Diseases, University of Edinburgh, Easter Bush, Roslin, Midlothian, EH25 9RG, UK; 2 Centre for Infectious Diseases, University of Edinburgh, Ashworth Laboratories, King's Buildings, West Mains Road, Edinburgh, EH9 3JF, UK; 3 Scottish Agricultural College, Animal Health Group, Research Division, King's Buildings, West Mains Road, Edinburgh, EH9 3JG, UK; 4 Biomathematics & Statistics Scotland, King's Buildings, West Mains Road, Edinburgh, EH9 3JZ, UK; 5 Scottish *E. coli *O157 Reference Laboratory, Western General Hospital, Crewe Road, Edinburgh, EH4 2XU, UK; 6 Comparative Epidemiology & Informatics, Institute of Comparative Medicine, Faculty of Veterinary Medicine, University of Glasgow, Bearsden Road, Glasgow, G61 1QH, UK

## Abstract

**Background:**

*E. coli *O157 is a bacterial pathogen that is shed by cattle and can cause severe disease in humans. Phage type (PT) 21/28 is a subtype of *E. coli *O157 that is found across Scotland and is associated with particularly severe human morbidity.

**Methods:**

A cross-sectional survey of Scottish cattle farms was conducted in the period Feb 2002-Feb 2004 to determine the prevalence of *E. coli *O157 in cattle herds. Data from 88 farms on which *E. coli *O157 was present were analysed using generalised linear mixed models to identify risk factors for the presence of PT 21/28 specifically.

**Results:**

The analysis identified private water supply, and northerly farm location as risk factors for PT 21/28 presence. There was a significant association between the presence of PT 21/28 and an increased number of *E. coli *O157 positive pat samples from a farm, and PT 21/28 was significantly associated with larger *E. coli *O157 counts than non-PT 21/28 *E. coli *O157.

**Conclusion:**

PT 21/28 has significant risk factors that distinguish it from other phage types of *E. coli *O157. This finding has implications for the control of *E. coli *O157 as a whole and suggests that control could be tailored to target the locally dominant PT.

## Background

*Escherichia coli (E. coli) *O157 is a bacterial pathogen capable of causing potentially fatal gastrointestinal disease in humans. In 2004 there were a total of 918 laboratory confirmed infections in the UK [1,2]. 209 of these cases occurred in Scotland where the rate of infections per hundred thousand is consistently higher than in the rest of the UK[1]. *E. coli *O157 is shed by cattle, which are believed to be a major reservoir for human infections. Phage type (PT) 21/28 is a subtype of *E. coli *O157 that was first detected in Scotland in 1994 [3]. By 2003 this PT dominated Scottish clinical cases, accounting for roughly two thirds of human isolates [4]. PT 21/28 is of particular concern because of its association with severe morbidity. Haemolytic uraemic syndrome (HUS) is a potentially fatal complication of *E. coli *O157 infection. A recent survey of HUS cases in the UK and Ireland indicated that the risk of developing diarrhoea-associated HUS was significantly higher in children in Scotland infected with PT 21/28 than with the majority of other PTs [5].

The aims of this study were to use data collected during a survey of Scottish cattle farms to estimate the prevalence of PT 21/28, to investigate the association between PT and the count of *E. coli *O157 in faecal pats, and to identify risk factors for the presence of PT 21/28 at the farm level relative to other PTs.

## Methods

### Study Group and Sampling Protocol

The study group comprised 88 Scottish cattle farms on which the presence of *E. coli *O157 was identified during a larger cross-sectional survey of 481 farms conducted from February 2002 to February 2004. The 481 study farms were stratified into 6 regions, corresponding to the six Scottish animal health divisions recognised by the Scottish Executive Environment and Rural Affairs Department (SEERAD). Within each region, farms were sampled throughout the 2 year period in clusters of three. The principal farm in each cluster was selected at random and a list of the six farms nearest to it but within the same region was drawn up. Following consent to visit the principal farm, farms on the list of six were approached sequentially to identify a sampling cluster of three participating farms. Farms in the same cluster were visited on the same or contiguous days. On each farm, groups of store and finishing cattle were identified and sampled. The number of faecal pats sampled in each group was determined from the number of cattle in the group using a prescribed sampling schedule. For each group, sufficient pat samples were taken to ensure 90% probability of detecting shedding of *E. coli *O157 if on average 8% of animals were shedding in positive groups, with shedding distributed as seen in an earlier study commissioned by the Scottish Executive Environment and Rural Affairs Department. Faecal samples were refrigerated at 5°C. The majority were refrigerated within two hours of sampling, while a small number were held at ambient temperature before refrigeration on the day after sample collection.

### Laboratory Analysis

Within 48 hours faecal samples were examined by immunomagnetic separation (IMS) to detect the presence of *E. coli *O157 as described by Pearce et al., 2004 [6]. To estimate *E. coli *O157 counts, 1 g of faeces from each *E. coli *O157 positive sample was suspended in 9 ml of maximum recovery diluent (Oxoid Ltd, Basingstoke, UK) and 0.1 ml of suspension spread onto each of two CT-SMAC plates. Plates were incubated at 42°C for 24 hours. Typical non-sorbitol fermenting colonies were counted and tested using anti-*E. coli *O157 coated latex reagent (Oxoid Ltd.). The limit of accurate enumeration using this method was 100 colony forming units (CFU)g^-1 ^faeces [7]. One *E. coli *O157 isolate from each faecal sample was submitted to the Scottish *E. coli *O157 Reference Laboratory for phage typing [8] and screened for genes encoding the virulence factors verocytotoxin 1 (*vtx*_*1*_), verocytotoxin 2 *(vtx_*2*_) *and intimin (*eae*) [6].

### Classification of Farm Status and Questionnaire Survey

*E. coli *O157 was identified on 91 of the 481 farms in the full study. Due to an incomplete data set, three farms were excluded and 88 farms were included in this analysis. Laboratory data were binary coded to establish the probability of observing PT 21/28 presence on *E. coli *O157 positive farms. A farm was classified as positive for PT 21/28 if at least one *E. coli *O157 isolate from that farm was of this PT. Farms were classified as negative if all isolates from the farm were phage typed and none were PT 21/28. On each sampling visit a confidential questionnaire was administered by one of three individuals, to gather information on the farm environment, the types of livestock present, the farm location and the housing, feed and management of the sample groups. The number of *E. coli *O157 positive pats on each farm was recorded and analysed as a predictor variable.

### Risk Factor Analysis

Generalised linear modelling was carried out to identify variables associated with PT 21/28 presence. Initially, 109 variables were individually tested for association with PT 21/28 presence in a univariate generalised linear model (GLM) with a logistic link function. All variables with univariate likelihood ratio test p ≤ 0.3 were carried forward to a multiple variable analysis. The multiple variable model was constructed through forward selection. Variables were added to the null model in order of decreasing significance until no further variables were significant at p ≤ 0.05. At each step all variables were checked to ensure that they maintained significance at p ≤ 0.05. Finally, the multiple variable model was fitted as a generalised linear mixed model (GLMM) with sampling cluster fitted as the random effect to account for the clustering in sample design. The multiple variable GLM models constructed through forward selection were assessed using Hosmer & Lemeshow diagnostic plots [9]. To identify any problems with collinearity, variable coefficients from the multiple variable models were compared with the corresponding univariate GLM coefficient to look for marked changes in direction or magnitude of effect in the multiple variable models. All two-way interactions were also tested.

### Analysis of Bacterial Counts

*E. coli *O157 count data were categorised, and a contingency table comparing the counts of pat samples containing a PT 21/28 isolate to the counts of samples containing a non-PT 21/28 *E. coli *O157 isolate was constructed. The association between *E. coli *O157 type (PT 21/28 or non-PT 21/28) and count category was assessed using a chi-square test. All analysis was conducted using R (The R Foundation for Statistical Computing, 2004, Version 2.0.1 (2004-11-15), ISBN 3-900051-07-0).

## Results

A total of 509 *E. coli *O157 isolates from 88 farms were phage typed and tested for the presence of common virulence genes. Isolates of 11 different PTs were found but there was a clear predominance of PT 21/28 (50.5%) (Table 1). PT 21/28 was present on 50/88 *E. coli *O157 positive farms (56.8%, 95% CI = 45.8, 67.4%). The presence of *E. coli *O157 isolates of only one PT was recorded on 70 of the 88 farms, including 37 farms on which only PT 21/28 was found. PT 21/28 was also identified on 13 of the 18 farms on which isolates of two or three different PTs were present. All but 3 *E. coli *O157 isolates carried the *eae *gene encoding intimin, 98% carried the *vtx*_*2 *_verocytotoxin gene and only 4 isolates carried neither the *vtx*_*1 *_or *vtx*_*2 *_gene.

One hundred and nine variables were tested for association with PT 21/28 presence and 49 were significant at p ≤ 0.3. No variables concerning housing, the use of manure, the use of slurry, or cattle feed were significant. In the final multiple variable GLMM three fixed effects were found to be formally statistically significant (Table 2). No interaction terms were significant.

Farms on which the water supply to the farmhouse was from the mains (n = 60) were significantly less likely to be infected with PT 21/28 than farms that had a private water supply (n = 28) (OR = 0.0002). This variable refers to farmhouse water supply rather than cattle water supply. However, 55 of 60 farms with a mains supply to the farmhouse also had a mains water supply for their cattle and there is highly significant correlation between the farmhouse and cattle water sources (χ^2 ^= 44.5, df = 1, p < 0.0001). The presence of PT 21/28 on a farm was positively associated with the number of pat samples from the farm that were positive for *E. coli *O157 (OR = 1.36). The Y coordinate of the farm was associated with the presence of PT 21/28, such that more northerly farms were more likely to be infected with PT 21/28 (OR = 1.0014). The odds ratio values for the number of *E. coli *O157 positive samples and Y coordinate represent a change in the odds of PT 21/28 presence per unit increase in the predictor variables – increases of a single positive pat sample and 1 km northing respectively.

**Figure 1:**
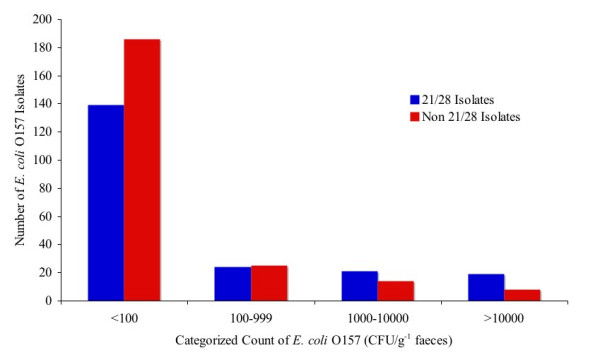
Comparison of the categorised count of *E. coli *O157 for samples containing PT 21/28 and non-PT 21/28 *E. coli *O157 isolates.

Analysis of *E. coli *counts revealed that PT 21/28 isolates were significantly associated with categorized *E. coli *O157 counts (χ^2 ^= 10.69, df = 3, p = 0.01). *E. coli *O157 positive pat samples containing a PT 21/28 isolate were underrepresented in the smaller count categories and overrepresented in the larger count categories, as compared to samples containing a non-PT 21/28 isolate (Figure 1).

## Discussion

Approximately half (50.5%) of the *E. coli *O157 isolates analysed in this study were PT 21/28, and this PT was present on 56.8% of *E. coli *O157 positive study farms. Three statistically significant risk factors specific to PT 21/28 presence have been identified. Farms on which the farmhouse water was supplied from the mains were significantly less likely to be infected with PT 21/28 than farms that had a private water supply. The presence of *E. coli *O157 in a cattle water supply is positively associated with the presence and extent of faecal shedding (PT not specified) [10-12]. The association seen here between PT 21/28 presence and a private water supply suggests that waterborne transmission may play a particularly important role in the transmission of PT 21/28 *E. coli *O157. The latitude of the farm is also associated with the likelihood of PT 21/28 presence, such that farms located further north are more likely to have cattle shedding PT 21/28. Investigation of climatic and farm management factors that may vary with latitude, and specific spatial analyses of the distribution of PT 21/28 isolates may identify further potentially important risk factors for PT 21/28 presence.

The analysis of the bacterial count data reveals that PT 21/28 isolates are associated with larger *E. coli *O157 counts (Figure 1). Only one *E. coli *O157 isolate from each pat sample was characterised, but pilot studies indicated that a single isolate type tends to dominate within individual pat samples. The association between the presence of PT 21/28 and an increased number of *E. coli *O157 positive pat samples is difficult to interpret and could simply reflect an increased likelihood of finding any phage type when a greater number of *E. coli *O157 isolates are tested. However, on 76% (38/50) of the farms on which PT 21/28 was present, PT 21/28 was the only phage type identified, even though some of these farms had as many as 25 positive isolates. Previous studies have identified an association between the presence of supershedding cattle on a farm and low level shedding in other individuals [13].

## Conclusion

This study was carried out using a limited data set including 88 farms on which *E. coli *O157 was present. However, this work has led to a determination of the prevalence of PT 21/28 on *E. coli *O157 positive Scottish cattle farms, the identification of a significant association between PT 21/28 presence and larger counts of *E. coli *O157 in faecal pats and the identification of statistically significant risk factors specific to PT 21/28 *E. coli *O157. If different phage types of *E. coli *O157 have different risk factors and ecology, the concentration of future research efforts upon particular subtypes may allow the identification of more specifically relevant risk factors. PT 21/28 has emerged relatively recently in Scotland, effectively superseding previously dominant phage types. By recognising the heterogeneity within the total *E. coli *O157 population and concentrating future research upon more specific sub-populations such as PT 21/28 it may be possible to gain more accurate and informative data about the ecology and control of *E. coli *O157 overall.

## Competing interests

The author(s) declare that they have no competing interests.

## Authors' contributions

JH participated in data analysis and preparation of the manuscript. MECT participated in the analysis, interpretation and presentation of findings. MP and DM collected the farm data and advised on the interpretation of findings. LA conducted laboratory analysis and advised on the interpretation of findings. IJM participated in the analysis and interpretation of findings. DF carried out the laboratory analysis of bacterial counts. CL and GG coordinated data collection and analysis. MEJW participated in the interpretation of findings and preparation of the manuscript. All authors read and approved the final manuscript.

## Figures and Tables

**Table 1:** Phage types and *vtx *gene possession of the 509 *E. coli *O157 isolates from the 88 *E. coli *O157 positive study farms.

**Phage Type**	**Number of *vtx1 *Positive Isolates**	**Number of *vtx2 *Positive Isolates**	**Total Number of Isolates**		**% of Characterized Isolates**
21/28	0	257	257	(50)	50.5
32	0	85	85	(26)	16.7
8	48	47	51	(9)	10.0
2	0	50	50	(8)	9.9
34	0	22	22	(1)	4.3
33	0	7	7	(1)	1.4
4	0	6	6	(3)	1.2
31	0	2	5	(2)	1.0
49	0	2	2	(2)	0.4
54	0	2	2	(1)	0.4
14	0	1	1	(1)	0.2
RDNC*	1	18	18	(4)	3.5
Untypable	0	0	3	(2)	0.6

Total Isolates	49	499	509		

Figures in brackets indicate the number of farms on which the isolates of each phage type were found. * Phages Reacted but Did Not Conform to any known pattern

**Table 2:** Summary of the multiple variable mixed model of the presence of PT 21/28 with sampling cluster fitted as the random effect

**Variable**	**Coefficient**	**S.E.**	**p-value**	**OR**	**95%CI**
Intercept	-5.21	4.44	-	-	-
Mains water to the farmhouse	-8.56	1.25	< 0.0001	0.0002	1.64E^-5 ^: 2.22E^-3^
Number of positive samples	0.30	0.07	0.0002	1.36	1.19 : 1.56
Y coordinate (1 km)	0.014	0.006	0.026	1.014	1.0027 : 1.026

OR = odds ratio, 95% CI = odds ratio ± 1.96SE. Random effect variance components: sampling cluster = 23.25, residual = 0.11.

## References

[B1] Health Protection Scotland Laboratory isolates of *Escherichia coli *O157 in humans reported to HPS, 1995–2004.

[B2] Health Protection Agency Vero cytotoxin-producing *E. coli *O157. Strain examined by LEP. Isolations from Humans. England and Wales, 1982–2004. http://www.hpa.org.uk/infections/topics_az/ecoli/O157/data_ew.htm.

[B3] Sharp JCM, Reilly WJ, Coia JE, Curnow J, Synge BA (1995). *Escherichia coli *O157 infection in Scotland: an epidemiological overview, 1984–94. PHLS Microbiology Digest.

[B4] Locking M, Allison L, Rae L, Hanson MF (2004). VTEC in Scotland 2003: enhanced surveillance and reference laboratory data. SCIEH Weekly Report.

[B5] Lynn RM, O'Brien SJ, Taylor CM, Adak GK, Chart H, Cheasty T, Coia JE, Gillespie IA, Locking ME, Reilly WJ, Smith HR, Waters A, Willshaw GA (2005). Childhood Hemolytic Uremic Syndrome. United Kingdom and Ireland. Emerging Infectious Diseases.

[B6] Pearce MP, Jenkins C, Vali L, Smith AW, Knight HI, Cheasty T, Smith HR, Gunn GJ, Woolhouse MEJ, Amyes SGB, Frankel G (2004). Temporal Shedding Patterns and Virulence Factors of *Escherichia coli *Serogroups O26, O103, O111, O145 and O157 in a Cohort of Beef Calves and Their Dams. Applied and Environmental Microbiology.

[B7] Pearce MP, Fenlon D, Low JC, Smith AW, Knight HI, Evans J, Foster G, Synge BA, Gunn GJ (2004). Distribution of *Escherichia coli *O157 in Bovine Fecal Pats and Its Impact on Estimates of the Prevalence of Fecal Shedding. Applied and Environmental Microbiology.

[B8] Khakria R, Duck D, Lior H (1990). Extended phage-typing scheme for *Escherichia coli *O157:H7. Epidemiology & Infection.

[B9] Hosmer DW, Lemeshow S (1989). Applied logistic regression.

[B10] Shere JA, Bartlett KJ, Kaspar CW (1998). Longitudinal Study of *Escherichia coli *O157:H7 Dissemination on Four Dairy Farms in Wisconsin. Applied and Environmental Microbiology.

[B11] Sargeant JM, Sanderson MW, Smith RA, Griffin DD (2003). *Escherichia coli *O157 in feedlot cattle feces and water in four major feeder-cattle states in the USA. Preventive Veterinary Medicine.

[B12] Sargeant JM, Sanderson MW, Smith RA, Griffin DD (2004). Associations between management, climate and *Escherichia coli *O157 in the faeces of feedlot cattle in the Midwestern USA. Preventive Veterinary Medicine.

[B13] Low CJ, McKendrick IJ, McKechnie C, Fenlon D, Naylor SW, Currie C, Smith DGE, Allison L, Gally DL (2005). Rectal carriage of Enterohemorrhagic *Escherichia coli *O157 in Slaughtered Cattle. Appl Environ Microbiol.

